# A roadmap to using historical controls in clinical trials – by Drug Information Association Adaptive Design Scientific Working Group (DIA-ADSWG)

**DOI:** 10.1186/s13023-020-1332-x

**Published:** 2020-03-12

**Authors:** Mercedeh Ghadessi, Rui Tang, Joey Zhou, Rong Liu, Chenkun Wang, Kiichiro Toyoizumi, Chaoqun Mei, Lixia Zhang, C. Q. Deng, Robert A. Beckman

**Affiliations:** 1 Data Science & Analytics, Bayer U.S. LLC, Pharmaceuticals, 100 Bayer Boulevard, Whippany, NJ 07981 USA; 2 Center of Excellence, Methodology and Data Visualization, Biostatistics Department, Servier pharmaceuticals, 200 Pier Four Blvd, Boston, MA 02210 USA; 3 Biometrics, Xcovery LLC, Pharmaceuticals, 11780 U.S. Hwy 1 N #202, Palm Beach Gardens, FL 33408 USA; 4 Bristol-Myers Squibb, 300 Connell Drive, 7th, Berkeley Heights, NJ 07922 USA; 5 Biostatistics department, Vertex Pharmaceuticals, Inc, 50 Northern Avenue, Boston, MA 02210 USA; 6 Biometrics, Shionogi Inc, 300 Campus Drive Florham Park, Florham Park, NJ 07932 USA; 7 Institute for Clinical and Translational Research, Department of Biostatistics and Medical Informatics, School of Medicine and Public Health, University of Wisconsin-Madison, Madison, WI 53726 USA; 8 Scipher Medicine, 260 Charles St Path, Waltham, MA 02453 USA; 9 United Therapeutic Corp, Research Triangle Park, Durham, NC 27709 USA; 10 Lombardi Comprehensive Cancer Center and Innovation Center for Biomedical Informatics, Georgetown University Medical Center, Washington, DC 20007 USA

**Keywords:** Historical control, Clinical trial, Simulation, Sensitivity analysis, Rare disease, Pediatric indication, Real world data, Real world evidence

## Abstract

Historical controls (HCs) can be used for model parameter estimation at the study design phase, adaptation within a study, or supplementation or replacement of a control arm. Currently on the latter, there is no practical roadmap from design to analysis of a clinical trial to address selection and inclusion of HCs, while maintaining scientific validity. This paper provides a comprehensive roadmap for planning, conducting, analyzing and reporting of studies using HCs, mainly when a randomized clinical trial is not possible. We review recent applications of HC in clinical trials, in which either predominantly a large treatment effect overcame concerns about bias, or the trial targeted a life-threatening disease with no treatment options. In contrast, we address how the evidentiary standard of a trial can be strengthened with optimized study designs and analysis strategies, emphasizing rare and pediatric indications. We highlight the importance of simulation and sensitivity analyses for estimating the range of uncertainties in the estimation of treatment effect when traditional randomization is not possible. Overall, the paper provides a roadmap for using HCs.

## Background

Scientists may decide to use only ***historical control data*** to fully or partially replace a concurrent control. Especially when there are either ethical concerns in recruiting patients for control arms in life threatening diseases with no credible control arm, it is clear that an alternative source of control data is essential. Secondly, challenges in developing underserved indications [1] may be partially ameliorated by historical controls, making drug developers more likely to invest, as programs will be somewhat more cost-effective. In particular, by reducing required patient numbers, historical controls may make enrollment of rare disease trials more feasible.

***Historical data*** including, but not limited to, historical controls can also be used either at the study design phase for refining parameter estimations or for adaptation within a study [2]. An interesting example of the latter is the use of maturing phase II data to govern adaptations at the interim analysis in a phase III study [3]. In a phase III study with time to event (TTE) endpoints that take time to develop, interim analyses are usually performed using data from within the phase 3 study. In this conventional approach, one must either perform the interim adaptation based on a very small amount of immature data concerning the slowly developing definitive endpoint, or rely on a rapidly developing surrogate endpoint that correlates imperfectly with the definitive endpoint.

Alternatively, one may use historical data from a previously conducted phase II study to govern the adaptation within the phase 3 study, which would then allow the adaptation to be governed by a larger amount of mature definitive endpoint data [2]. This example is one of many of using historical data for study design or adaptation. The example differs from the other examples in this paper in two important respects: First, the example involves use of historical data of treatment effect, not just a historical control; Second, the historical data, are not part of the final confirmatory evidence package submitted for approval, which is limited to data within the phase 3 study only.

Similar to any other approaches, use of HCs is not always the best approach as there are pros and cons associated with it. Thus, researchers should plan for simulation and thorough sensitivity analysis, and account for and interpret the results with caution. When HCs are part of the final confirmatory package, this paradigm should be reserved for carefully selected products and clinical conditions in which RCTs are not practical [1, 4, 5]. Using HCs requires a robust justification and involvement of regulatory bodies early on for non-RCT phase III clinical trials [6].

## Resources of HCs

In general, one is searching for HC data that is as similar as possible to the patients being enrolled in the study of interest. Similarity of patients should go beyond the standard baseline characteristics to include other considerations such as the healthcare environment, background therapy, progress in the standard of care, psychological effects, etc. Data may come from different sources with a variety of structure and quality, which results in different biases and concerns for use of different types of HCs. This must be accounted for when a decision is made to use HCs in a clinical trial [3, 7, 8]. Here we list main resources of HCs in the current practice and discuss the pros and cons using those resources and our recommendations.

### Real world data (RWD)

It can exist across a wide spectrum, ranging from observational studies within an existing database to studies that incorporate planned interventions with or without randomization at the point of care [9]. Medical charts, published data of off-label use, registries and natural history studies are all examples of real world data. In addition, RWD includes any data relating to patient health status and/or the delivery of health care routinely collected from a variety of sources [10]. Studies leveraging RWD can potentially provide information on a wider patient population that cannot be obtained through classic clinical trials. An existing RWD source, however, may have inherent biases that could limit its value for drawing causal inferences between drug exposure and outcomes. To mitigate the potential bias, a study protocol and analysis plan should be created prior to accessing, retrieving, and analyzing RWD, regardless of whether the RWD are already collected retrospectively or if they are to be collected prospectively. Protocols and analysis plans for RWD should address the same elements that a classic clinical trial protocol and statistical analysis plan would cover. When considering a prospective study design, one should consider whether RWD collection instruments and analysis infrastructure are sufficient to serve as the mechanism for conducting the study or is it possible to modify them for such a purpose. Ultimately, if the sources of bias can be mitigated, RWD collected using a prospective study design may be used to generate or contribute to the totality of the evidence regarding the control arm. The increased use of electronic data systems in the healthcare setting has the potential to generate substantial amounts of RWD. Because of its nature, the quality of RWD can vary greatly across different data type and sources. For the relevance and reliability of RWD, RWD sources and resultant analysis please see Appendix 1. Below some of these resources are discussed further.

#### Medical chart

It is a complete record of a single patient’s medical history, clinical data and health care information at a single institution, maybe supplemented by prior institutions, which is usually incomplete. It contains a variety of medical notes made by a physician, nurse, lab technician or any other member of a patient’s healthcare team, as well as laboratory, diagnostic and therapeutic procedures data.

Even though the majority of older paper charts have been replaced by digital electronic heath records (EHR), the structure and quality of ***data is still challenging to work with*** due to frequent missing data, inaccuracies, and difficulties in extracting unstructured free text information in medical notes, the most informative component.

Even though concurrent patients can be selected, there is a possibility of introducing a ***patient selection bias***, as it has shown in several studies that patients from clinical trials tend to have better outcomes than those seen in routine practice. This could be due to the variability of quality of routine practice in different regions [11–13]. Regardless of available matching techniques, it must be borne in mind that the characteristic that clinicians use for patient selection when performing clinical trials are subtle and challenging to quantify. ***In order to avoid this bias***, it may be advisable to consider a **pragmatic clinical trial,** i.e. a trial in which the active arm data are also collected in a real world practice setting. This increases the generalizability of the results to the real world at the expense of decreased accuracy, increased missing data, and the need to strictly minimize study complexity.

***In the cases of ultra-rare diseases***, medical charts have been used as the basis for drug approval, e.g. CARBAGLU (Carglumic Acid) for the treatment of the deficiency of the hepatic enzyme N-acetylglutamate synthase (NAGS), the rarest of the Urea Cycle Disorders (UCDs), affecting fewer than 10 patients in the U.S. at any given time and fewer than 50 patients worldwide. This drug was approved in March 2010 based on a medical chart case series derived from fewer than 20 patients and comparison to a historical control.

#### Patient registry

It is an organized system that uses observational methods to collect uniform data on specified outcomes in a population defined by a particular disease, condition or exposure. At their core, registries are data collection tools created for one or more predetermined scientific, clinical, or policy purposes. Data entered into a registry are generally categorized either by diagnosis of a disease (disease registry) or by drug, device, or other treatment (exposure registry). There is also the option of using only data entered into the registry concurrently. This “***concurrent registry***” option minimizes the impact of time dependent covariates, such as improvement in supportive care for the condition of interest, as well as stage migration, the process by which increasing sensitivity of diagnostic techniques leads to classification of less severely affected patients for a given category over time [14, 15].

***The interoperability of registries*** is dependent upon the use of data standards. The quest for registry standards is complicated by the number of different registries, the variety of purposes that they serve, and the lack of a single governor of registries. ***Data standards*** are consensual specifications for the representation of data from different sources or settings. Part of the challenge for standards observance is the reality that often any given individual organization or registry project perceives little immediate benefit or incentive to implement data standards. Standards become vitally important, when data is being exchanged or shared, often benefiting a secondary user. Standardized data include specifications for data fields (variables) and value sets (codes) that encode the data within these fields.

Even with standardization, there is a good chance of frequent ***missing data*** in registries, as the culture of recording evidence and information in current practice is not comparable to what has been mandated in clinical trials. Appendix 2 summarizes the recommendation by Clinical Trials Transformation Initiative (CTTI) for evaluating an existing registry [15–17].

#### Natural history (NH) trial

NH trials track the natural course of a disease to identify demographic, genetic, environmental, and other variables that correlate with the disease and its outcomes in absence of a treatment [18, 19]. However, these studies often include patients receiving the current standard of care, which may postpone the disease progression. ***They differ from registries*** in that they can be designed specifically to collect comprehensive and granular data in an attempt to describe the disease, which may or may not be present to varying degrees in a registry. These trials are usually designed to address the special problems of rare disease clinical trial designs, where there may be little pre-existing information about natural history and preferred endpoints for study. The trials are uncontrolled and non-interventional. As such, existing NH trials are also a valuable source of HC.

If there are ***no pre-existing NH studies available***, the initiation of such studies has been recommended in parallel with early stages of drug development including preclinical, prior to the initiation of interventional trials [20]. Inclusion criteria of natural history studies should be broad, to allow characterization of the heterogeneity of the disease and the effect of covariates on outcome. However, in many cases rare diseases are rapidly progressive or fatal and affecting children. The ethical justification for conducting non-interventional trials prior to interventional trials under such circumstances may be questionable, if it delays the interventional trials.

Instead, ***alternative approaches*** that involve adaptive clinical trial designs are recommended [21, 22]. In some cases, sponsors may have more incentive to address the rare diseases directly instead of conducting a non-interventional study. For instance, it may be difficult to convince a sponsor to fund a NH study without proof of concept for the therapy it is developing. Despite these issues, NH studies have been used in some development programs to approve therapies successfully (Appendix 3).

### Completed clinical trials

***Completed clinical trials*** for the drugs with the same mechanism of action (MOA) or the same drug from previous phases of development are a great source of high-quality data, since they are generated in a controlled environment. The control (placebo or standard of care, or SOC) arm of completed clinical trials can be treated as a source of control data for an indication [23, 24].

## Current use of HCs in pivotal clinical trials and the regulatory outcome

Even though our focus is not limited to pivotal studies, we illustrate the current use of HCs for approvals by regulatory agencies (Table 1). The majority of confirmatory clinical trials using historical controls have indications in rare diseases, where there are no approved therapies for SOC. Other common applications for HC in the confirmatory setting are: medical devices [9], label expansion, pediatric indications, and small populations such as biomarker subgroups. ICH and FDA E10 guidance accept the use of external controls as a credible approach only in exceptional situations, in which either the effect of treatment is dramatic and the usual course of the disease highly predictable and the endpoints are objective and the impact of baseline and treatment variables on the endpoint is well characterized. In the majority of cases listed in Table 1, the treatment had either a large effect size and/or addressed a life-threatening disease with no treatment options, to be considered. However, the second example was not an exceptional case like the others. It was a phase III trial that used data from Phase II studies to determine the null hypothesis. The approvals of the drugs have been based on the totality of benefit-risk assessments, involving a qualitative judgment about whether the expected benefits of a product outweigh its potential risks. For example, EXONDYS 51 (eteplirsen) that treats duchenne muscular dystrophy was approved, although initially the agency raised doubts about results from small sample size of 12-patients and the advisory panel voted against the approval.

**Table 1:** Examples of HCs used in Clinical Trials for Approvals by Regulatory Agencies

Drug Candidate	Carglumic Acid or CARBAGLU	Protein C Concentrate (Human) or CEPROTIN	Lepirudin or REFLUDAN	Antithrombin [Recombinant] or ATRYN
Indication	Hyper-ammonaemia	Protein C deficiency	Immunologic type of Heparin-associated thrombocytopenia	Hereditary anti-thrombin deficient patients
Prevalence of the disease	< 10 patients (US) < 50 (worldwide)	< 20 patients (US)	N/A	N/A
Other treatments	No	No	No	No
Approved	2010	2007	1998	2009
Division	FDA-CDER	FDA-CDER	FDA-CDER	FDA-CDER
Study Design	Retrospective Case Series	N/A	Uncontrolled study	Two prospective, single-arm, open-label studies
Number of subjects	23 subjects	18 subjects	Two studies with 39 and 33 Subjects	31 subjects
Endpoint	Ammonia levels	Response for thromboembolic events	Platelet count recovery	Incidence of thromboembolic events
Type of HC	Natural history data	N/A	Registry data which PI established	Prospectively designed retrospective chart review
Source of the HC	Subject at baseline	N/A	Subjects not treated with recombinant hirudin	
Size of HC	23 subjects	21 subjects	91 subjects	35 Subjects
Method of application	Descriptive statistics	Descriptive Statistics	Direct comparison	Matching
Note			HC was used only for secondary endpoint due to the less availability of platelet data	
**Drug Candidate**	**Anagrelide or AGRYLIN**	**Alglucosidase Alfa or MYOZYME/LUMIZYME**	**Miglustat or ZAVESCA**	**Blinatumomab or BLINCYTO**
Indication	Reduction of the elevated platelet count, thrombosis and ameliorate-associated symptoms.	Pompe disease	Type I Gaucher disease	Adults relapsed/refractory Acute Lymphoblastic Leukaemia
Prevalence of the disease	N/A	1/40,000 live births	1/100,000	1–2/100,000 adults
Other treatments	No	No	Available	No
Approved	1997	2006	2003	2017
Division	FDA-CDER	FDA-CDER	FDA-CDER	FDA-CDER
Study Design	Self-controlled study	RCT with two dose groups	Self-controlled study	Single arm trial
Number of subjects	About 300 subjects	18 (9 subjects per dose group)	Three studies include 28, 18 and 36 subjects	189 subjects
Endpoint	N/A	Invasive ventilator-free survival and survival rate	Percentage change from baseline in liver organ volume	Complete remission
Type of HC	Natural history data at baseline	Natural history data (Cross-sectional NH)	Natural history data at baseline	Several kinds of data
Source of the HC	Subject at baseline	Natural history data	Subject at baseline	Investigator database
Size of HC	About 300 subjects	62 subjects	82 subjects	1112 subjects
Method of application	N/A	Direct comparison for non-inferiority inference.	Descriptive Statistics	HC was used to show the validity of efficacy threshold by meta analytic approach.
Note		Historical data collected over a 20-year span and time trend observed.		For EMA, analysis with propensity score was also performed.
**Drug Candidate**	**Arsenic trioxide or TRISENOX**	**Eteplirsen or EXONDYS 51**	**Elbasvir and Grazoprevir or ZEPATIER**	**Nitisinone or ORFADIN**
Indication	Acute promyelocytic leukaemia	Duchenne muscular dystrophy (DMD)	Treatment of Chronic Hepatitis C genotypes 1, 4 or 6 in adults	Hereditary Tyrosinemia 1
Prevalence of the disease	6/10,000,000 per year	20 / 100,000	N/A	1/100000
Other treatments	Yes	No	Newly available during the development	No
Approved	2010	2016	2016	2002
Division	FDA-CDER	FDA-CDER	FDA-CDER	FDA-CDER
Study Design	Single arm trial	Placebo controlled study followed by extension study	Parallel or single arm study	Single arm study
Number of subjects	40 subjects	12 subjects	1294 subjects from 3 studies	207 subjects
Endpoint	Complete remission	Controlled trial: change from baseline of dystrophin positive fibers Extension study: 6MWT	Sustained virologic response	Survival
Type of HC	Registry data	Registry data	Previous clinical trial data	Registry data
Source of the HC	Hospital stored data	Matching from 2 DMD patient registries	Previous clinical trial data	Survey result
Size of HC	27 subjects	13 subjects	Depending on the trials	108 subjects
Method of application	Just showed as reference	Direct comparison	One sample testing. HC was used for efficacy threshold.	Just showed as reference
Note		Initially agency and advisory panel voted against the approval	Not a rare disease.	Large improvement against historical data
**Drug Candidate**	**Sodium Ferric Gluconate Complex or FERRLECIT**	**Sebelipase Alfa or KANUMA**	**Asfotase Alfa or STRENSIQ**	**Cerliponase Alfa or BRINEURA**
Indication	Iron deficiency anemia undergoing chronic hemodialysis	Lysosomal acid lipase (Wolman disease)	Hypophosphatasia	late infantile neuronal ceroid lipofuscinosis type 2
Prevalence of the disease	N/A	1/500,000	1/100,000	1/100,000
Other treatments	Yes	No	No	No
Approved	2001	2015	2015	2017
Division	FDA-CDER	FDA-CDER	FDA-CDER	FDA-CDER
Study Design	Multiple dose historical control study	Historical control study	Single-arm	Single arm trial
Number of subjects	88 subjects from 2 dosing groups	9 subjects	70 subjects from 2 studies	22 subjects
Endpoint	Change in Hemoglobin	Time to Death	Overall survival	Response rate
Type of HC	Registry data	Natural history data	Natural history data	Natural history data
Source of the HC	Subjects with oral Iron	Retrospective clinical chart reviews	Retrospective clinical chart reviews	Natural history cohort
Size of HC	25 subjects	21adjudicated as appropriate for comparison	48 subjects	42 subjects
Method of application	Direct comparison	Direct comparison by survival analysis	Direct comparison by survival analysis	Matched analysis with HC
Note	Another study also supported the efficacy			

## Minimize disadvantages of using HC in clinical trials [25, 26]

As discussed in Resources of HCs section, there may be potential biases in using HCs that have to be accounted for. In classical designs, even though randomization and blinding techniques do not guarantee the complete elimination of unknown confounders, they reduce the chance of bias and dissimilarity among the arms of a clinical trial. In a non-randomized study, an external control group is identified retrospectively, which potentially could lead to a ***selection bias*** or a ***systematic difference among groups*** that could affect the final outcome. These biases could come from dissimilarity in a wide range of factors: patient demographics may vary among different population; time trends bias may occur when a control arm is chosen from patients who were observed at some time in the past, or for whom data are available through medical records; the SOC treatment and concomitant medications may have improved over the years; the severity of actual disease associated with a particular stage designation may have decreased due to more sensitive diagnostic technologies (stage migration). For example, the medical devices and the accuracy and precision of measurements may have improved. It has been shown that untreated patients in HCs have worse outcomes than a current control group in a randomized trial [27, 28], possibly due to more stringent inclusion and exclusion criteria in the trial, subtle selection bias, as well as improvement in medical care and potential increased access to care. Moreover, ***assessment bias*** or ***the lack of blinding*** of the investigator, patients or healthcare personnel may also affect the evaluation of subjective endpoints.

The literature is mostly focused on choosing an HC that matches with the currently designed clinical trials and reducing bias in the analysis. We recommend a different and more fundamental solution. If possible, start with choosing a high-quality HC for the indication and design the current study as closely as possible to the selected HC. The recommendations are consistent with Pocock’s Criteria published in 1976 [29]: Choosing an HC with the same**:** a) Inclusion/Exclusion criteria, b) type of study design, c) well-known prognostic factors, d) study quality, and e) treatment for the control group in a recent previous study in the design; and finally either use a concurrent HC or adjust for biases such as time dependent biases at the analysis step to the extent feasible. For example, placebo control groups of concurrent clinical trials are preferred if possible. If a placebo group is unethical, consider selecting trials with active control groups that received the same SOC treatment and design the trial as an add-on design using the same SOC, provided there is no pharmacological antagonism between the experimental therapy and the SOC and there is an acceptable toxicity profile of the combination. Otherwise, the rest of this paper provides guidance regarding selecting HCs for replacement or augmentation of a control arm for an existing clinical trial design, in case the existing clinical trial cannot be redesigned to match closely with the selected HCs.

It is of primary importance to improve the quality and usability of the data and subsequently increase the feasibility of using HCs in clinical trials. Thus, we recommend using standards as much as possible in HC data collections. A standard has been developed by the Clinical Data Standards Interchange Consortium (CDISC) [30] for regulated drug development submissions and drug safety. However, there are still some challenges.

Missing data can have a substantial impact on registry findings, as well as any clinical or observational study. It is important to take steps throughout the design and operational phases to avoid or minimize missing data; understanding the types of and reasons for missing data can help guide the selection of the most appropriate analytical strategy for handling the missing data, or the potential bias that may be introduced by such missing data.

Analysis methods can also help to minimize the disadvantages of using HCs in clinical trials. Several meta-analysis methods have been developed to analyze the historical data for epidemiology research, benchmarking and comparison of drug efficacy for a specific indication, developing the safety profile of a drug or device, marketing or hypothesis generation. Some of these methods are applied on summary data, and some require individual data. However, traditionally, this wealth of information has not been used directly in the analysis of the current data, which could increase the accuracy and precision of estimated relevant endpoints. The challenges for doing so are to determine: how much the HCs are similar to each other and to the current data; what is the optimal weight of the given HCs in the current analysis, in case of conflict between HCs or with the concurrent control data; and how to incorporate the HCs in the analysis with minimum bias.

Overall, these analysis methods can be classified as Frequentist and Bayesian. While Bayesian methods seem flexible as the amount of historical information to be combined with the study information can be adjusted based on the similarity among different available datasets (HCs and concurrent data), they have their own challenges. It’s noteworthy that the combination of information is not the same as “borrowing information”, since the inference is based on the combined information and not only on the study information with some borrowed information from HCs. However, all the listed methods use the “borrowing” term. There is no clear guidance of selection of specific priors and how dynamic borrowing is applied with control of the Type I error rate (i.e. the probability of making type I error) [31]. No one solution that works in all situations. Our recommendation is to perform simulations and to create a benchmark (see Appendix 4 and simulation section) to characterize each method for comparison to help with the decision-making process and, if using Bayesian methods, with the choice of proper prior [32, 33].

Generally, the historical information is discounted in recognition of the enhanced uncertainty when combining information from the past. **Dynamic borrowing** controls the level of borrowing based on the similarity of the historical control with the concurrent data. It borrows the most from past data when the past and current data are consistent and the least otherwise. If you have multiple HCs, the inter-trial variability is a measure of the suitability of the dataset for borrowing [34–40]. On the other hand, static borrowing sets a fixed level of borrowing, which is not determined by the level of consistency or inconsistency among the past and current datasets. The control for Type I error when borrowing can be addressed by simulation as discussed in the following sections. Recommendations for the use of simulations are given in the FDA draft guidance for adaptive designs released in 2018 [33].

### Frequentist approaches

Normally frequentist approaches may involve analyzing historical controls to estimate a threshold or parameter required for a simulation in a hypothetical scenario or balancing the population of different arms for a fair comparison. For details on frequentist approaches please refer to Appendix 4. The approaches can be further classified into two subgroups depending on whether individual data is available or only summary data. Mixed Treatment Comparison (MTC) or network analysis, Simulated Treatment Outcome (STC), or Matching-adjusted indirect comparison (MAIC) work with summary data [41]. If individual data is available, the **Propensity score (PS)** method is commonly used to match or stratify patients, to weigh the observations by the inverse probability of treatment, or to adjust covariates [42–44]. However, King et al., argues that matching based on propensity score often increases imbalance, inefficiency, model dependence, and bias [45]. They claim that “The weakness of PSM comes from its attempts to approximate a completely randomized experiment, rather than, a more efficient fully blocked randomized experiment. PSM is thus uniquely blind to the often large portion of imbalance that can be eliminated by approximating full blocking with other matching methods”.

**Threshold Crossing** introduces a new framework for evidence generation [1]. The HCs are used for estimation of futility and success thresholds. A single arm trial is conducted after obtaining these estimations. Bias-variance, a model suggested by Pocock [29], assumes a bias parameter with a specified distribution as a representative of the difference between past and present. **Test-then-pool** [37] compares the similarity of historical and concurrent control data with significance level α. If they are similar, it pools the HC and the concurrent control data; otherwise discards HC. In other words, it borrows nothing or all of the information from HCs.

**Meta-analytical approach** is a hierarchical modeling approach, which uses a data model and a parameter model to infer the parameter of interest (e.g. treatment effect). It is normally mentioned in the context of Bayesian approach, but it can be performed using a non-Bayesian approach [see Appendix 4]. **Adaptive Design** allows adaptations or modifications to different aspects of a trial after its initiation without undermining the validity and integrity of the trial [46]. This topic is out of the scope of this paper; however, one flavor of such design is **Adaptive Borrowing** that adjusts the recruitment of a concurrent control based on the evaluation of the similarity of HCs and concurrent control via several interim analyses. The Analysis can be performed using Bayesian and non-Bayesian approaches.

### Bayesian approaches

Most of the Bayesian methods aim for discounting or down-weighting the historical control [34]. **The power prior** method estimates an informative prior for the current study. The informative prior is the product of an initial prior (non-informative prior) and a likelihood function of the parameters of a given model given the HC data that is raised to a power between 0 and 1 (α_0_). The resulting ‘posterior’ is used as an informative prior for the current study. Alpha_0_ can be set to a fixed value (static borrowing) or can be set based on heterogeneity of HCs and the current data (dynamic borrowing). As mentioned before, **Adaptive Designs** can also use Bayesian approaches for the analysis of the data.

**Meta-analytic or hierarchical modeling** applies dynamic borrowing by placing a distribution of the degree of borrowing across current and historical controls, in which its variation is controlled by the level of the heterogeneity of the data. **Meta-analytic Combined (MAC)** and **Meta-analytic Predictive (MAP)** are two approaches of meta-analysis. It performs a meta-analysis of historical data and current trial data and infers the parameter of interest at the end of the new trial. MAC can be either a non-Bayesian or Bayesian approach using non-informative or vague prior to estimate the MAC model. MAP is a full Bayesian approach; it derives a “MAP prior” from historical data by derivation of a posterior distribution which expresses the information of all HCs. In the next step it combines the estimated “MAP prior” with the current trial data to get a posterior distribution of the parameter of interest (such as treatment effect) [See Appendix 4] [34]. The **Offset** approach, however, recommends using simulation and graphical tools to identify a range of plausible values for the true mean difference between historical and current control data. It argues that an estimate of bias based on comparison between the historical and new data gives little guidance on the value to be chosen for discounting the historical data. The offset approach claims that HC will be almost completely discounted, without a strong assumption regarding its relevance [47]. It should be pointed out that the differences between the current trial and HCs could be due to pure chance in sampling, specifically for rare diseases with small sample sizes. Ignoring HC information might lead to bias. Thus, additional scientific input is warranted.

### Sensitivity analysis

One crucial step in properly using HCs is performing sensitivity analyses, beyond what has been proposed by FDA for randomized clinical trials based on the primary analyses [48], to assess the robustness of the results. These analyses should interrogate any assumptions or criteria that could cause variation in the final result with regards to using HCs as a component of a clinical trial, i.e. the impact of cohort selection to address selection bias, the impact of missing data on key results, the method of matching patients with the historical controls, the level of borrowing using different priors and methods (power prior vs. mixed prior), the alteration of weighting factors when using any weighing schemes, the impact of exclusion of some historical controls when more than one is used, the unmet components of Pocock’s requirements for including a historical control, and the heterogeneity of historical controls with each other and with the concurrent control. Sensitivity analysis provides the full spectrum of potential truth in the presence of biases [49].

## Decision making

**Fig. 1:**
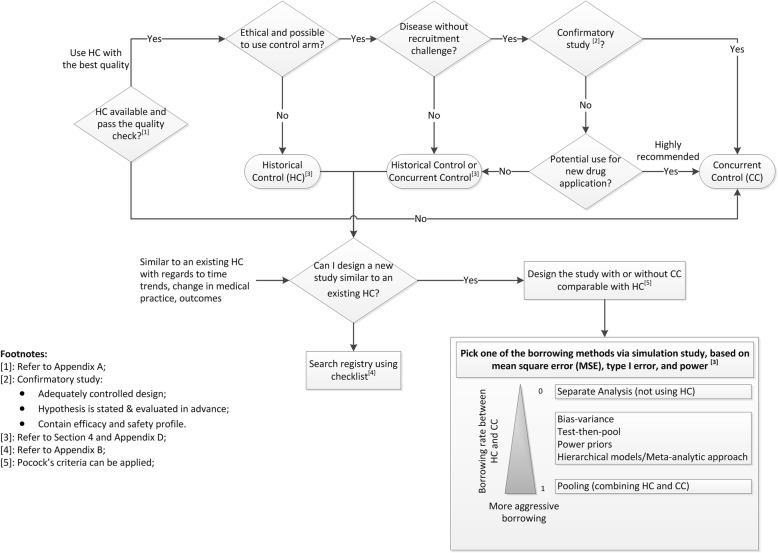
Decision Making Diagram for using HCs in Clinical Trials

Quantitative methods to extrapolate from existing information to support decision-making are addressed in a recent EMA draft guidance [50]. Here we propose a decision making tool (Fig. 1: Decision Making Diagram for using HCs in Clinical Trials), a procedure for planning, conducting, analyzing and reporting of studies using HCs, or as a supplement to a concurrent control arm, while maintaining scientific validity.

## Simulation role

**Fig. 2:**
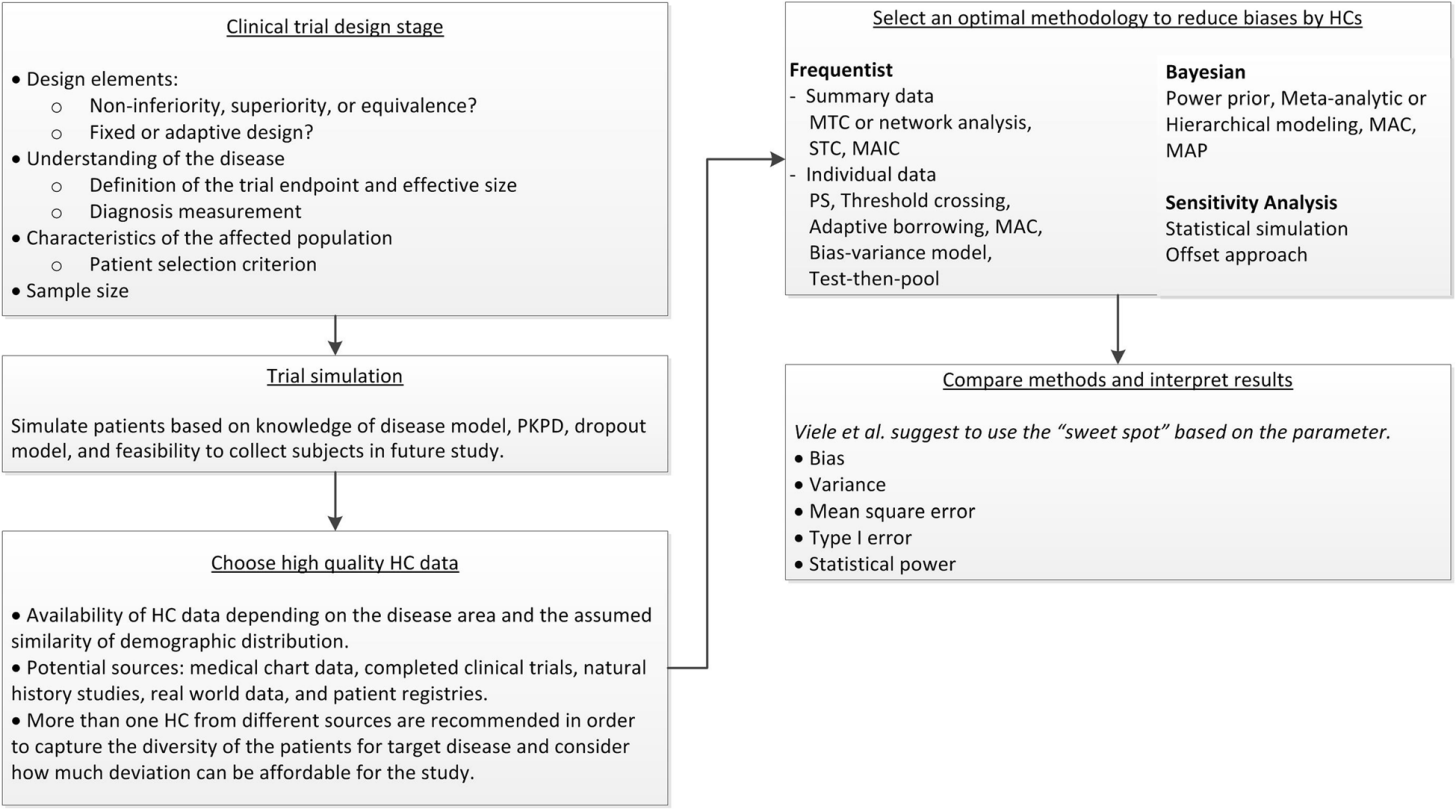
Clinical Trial using HCs Simulation Process

Clinical trials simulation plays a critical role in the drug development process by quantifying and evaluating design operating characteristics and possible decisions in the face of uncertainties [51]. It is also an important tool for selecting methods for bias control or for performing sensitivity analyses when HC is used in a trial. The operational characteristics of the design is of interest for Bayesian and frequentist approaches in order to assess the sensitivity of the decision-making process. For the Bayesian approach, a sensitivity analysis particularly for mismatch with regards to historical control, might be of interest. How to design, perform, and report such simulation studies deserve further attention and standardization [52]. Dejardin et al. demonstrate an example clinical trial simulation being applied to compare the performance among different Bayesian approaches for borrowing information from HC data [53]. The case study was a phase III comparative study of a new antibacterial therapy against *Pseudomonas aeruginosa*. Since the target population, patients with ventilator associated and hospital-acquired pneumonia, was a rare condition, HC became an attractive option. After identifying a non-inferiority combination design, mortality rate at 14 days as the primary endpoint, and maximum 300 subjects in total, the authors simulated trial data based on specific knowledge, e.g. mortality rate in randomized controlled studies. In order to reduce the impact of uncontrolled factors on HC validity, dynamic Bayesian borrowing was implemented to control the weights on use of compatible and incompatible HC data. Bayesian approaches with three different power priors were compared with regards to two outcomes: limited inflation in type I error and increase in power. The simulation predicted comparable performance among the three methods and therefore recommended the one with easiest implementation. This example illustrates best simulation practices and the benefits of applying them in the context of HCs. Best practices for simulation and reporting have recently been addressed by the DIA ADSWG [52]. The recent FDA draft guidance on Adaptive Designs also gives recommendations on clinical trial simulations [33] saying that simulations can be used to estimate not only basic trial operating characteristics, e.g. type I error probability and power, but also other relevant characteristics for more complex adaptive designs, such as expected sample size, expected calendar time to market or time to study completion, and bias in treatment effect estimates. The whole process is standardized in the diagram below (Fig. 2: Clinical Trial using HCs Simulation Process) that can be generalized to any clinical trial simulation.

Simulation can also be used for the comparison of different methods for choosing the “best” model for a specific design. I.e. One should compare the most effective area, “**sweet spots**”, among these methods, with lower MSE, lower type I error, and higher power compared to others when borrowing (using Frequentist or Bayes approaches) from historical controls [54]. Borrowing reduces type I error and increases the power of the study when the current control rate is close to the historical observed rate. This is intuitive as we are borrowing information nearly identical to the true current value. As the true current control diverges from the observed historical data, we can acquire reduced power (in one direction) and inflated type I error (in the other direction). Assessing the magnitude and relative likelihood of these costs in comparison to the possible benefits is the key issue in determining whether historical borrowing is appropriate in any given setting. In assessing a borrowing method in terms of MSE, type I error, and power, we can answer several questions. First, how broad the sweet spot is and how much power increases. The larger a region, where borrowing dominates the separate analysis, the more appealing the method will be. Second (and most important for possible confirmatory trials), where is the type I error and how much type I error inflation occurs? Third, how much of a power loss do we have when the true control is much lower than the historical data? These three regions (reduced power, sweet spot, and inflated type I error) create a decision problem in deciding how and whether to adopt borrowing.

**Fig. 3:**
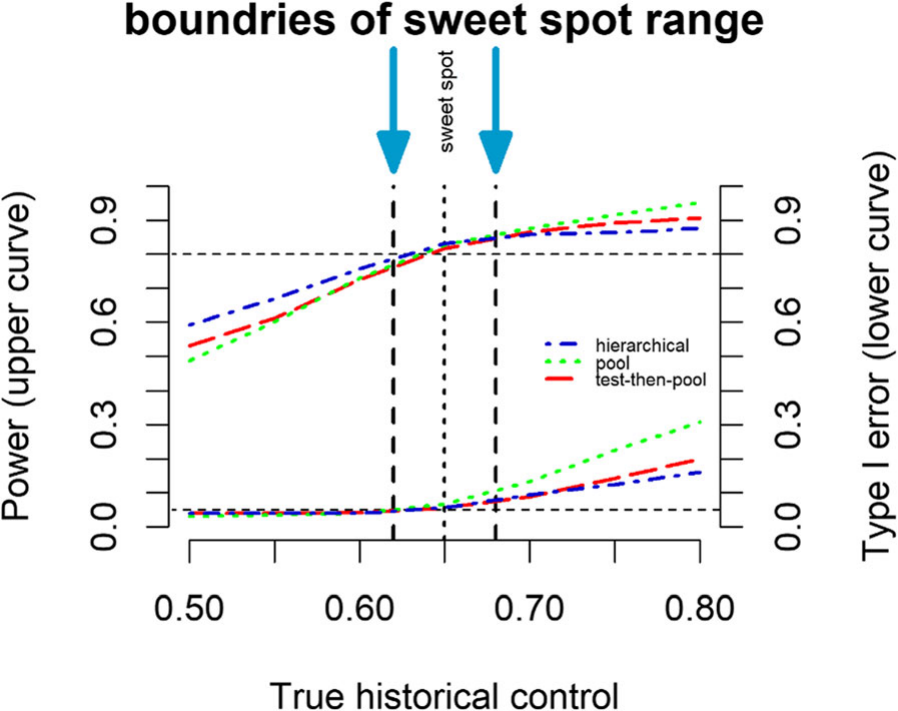
Visualization of Comparison of the “Sweet Spots” of Methods via Simulation

Fig. 3 shows a useful plot that can be generated via simulation to compare the sweet spot of different methods. This visualization shows type I error (lower curves, right axis) and power (upper curves, left axis) on the y-axis as a function of closeness of the event rate in historical data to the true control event rate on the x-axis for hierarchical models. Different line styles show different methods. Borrowing behavior tends to be ‘flatter’ for hierarchical models (blue curves), borrowing moderately over a long range, while still displaying dynamic borrowing (borrowing is reduced when the true control rate is far from the historical data). This moderate, long-range borrowing is also reflected in the type I error inflation that has a lower slope than other methods (although it still does reach reasonably high values). Generally, the sweet spot of improved type I error and higher power extends farther down (for values under 0.65) than other methods.

Finally, documented software (code) for all simulations should be made accessible to all stakeholders for reproducibility. However, if simulations are computationally intensive or the code is complex, it will be challenging to independently verify the results [52].

## Summary of recommendations

In Summary, for choosing a proper and high-quality HC for a credible result in a non-randomized clinical trial, the following steps are necessary in reduction of selection bias if the study design cannot be altered to mimic the selected HCs with regards to the time trend, population, change of SOC, logistics, and other risk factors. The important prerequisite for choosing a proper HC is to have a clear understanding of the disease, detailed characteristics of the affected population, precise definition of the endpoint and clear definition of the diagnosis in terms of what and how it has to be measured. It is also very important to know how to compare the measurements between the treatment groups while addressing interfering events such as rescue medication, drop-outs, death, non-adherence, and non-compliance. In other words, there has to be a very well-defined and precise scientific question or objective and analytic plan up-front before picking a historical control, so that the selection bias and ad-hoc analysis are minimized.

In general, choosing a simple endpoint reduces the complexity and subjectivity of the assessment and increases the precision of measurement and clarity of treatment efficacy. **First**, systematically review the literature for well-organized and maintained completed RCTs, Natural Histories, RWD, and registries for reproducibility of the existing evidence. Guidance provided from systematic reviews and meta-analyses could be helpful. Select an appropriately similar HC or HCs for the experimental setting. Explore and seek to understand the similarities and differences of different resources. It is recommended to choose more than one HC from different sources to capture the heterogeneity of treatment and the disease in different populations, which could give an estimation of treatment effect in the real world.

**Second**, select an applicable approach to account for biases in study design. Even with a well-thought design and proper selection of HCs, we may not be able to eliminate all the biases due to differences between the concurrent treatment group and HCs. Thus, we still need to consider adjusting for these biases by varying the level of borrowing information using Bayesian or frequentist methodologies (Section 7 and Appendix 4). If there are several HCs for example, one might give a different weight to HCs based on the variability or quality of the data. Adjust for selection bias by adjusting for patient’s covariance, if the detailed data are provided [55–59]. Otherwise, use summaries of baseline covariates. Use matching based on distance metrics (e.g. Euclidean distance) for direct standardization or use propensity score for stratification or covariate adjustment for indirect standardization in observational studies [60, 61]. Refer to Encyclopedia of Biostatistics for specialized methods for time-to-event endpoints [62]. Address the assessment bias due to the lack of blinding and randomization by recruiting patients receiving placebo or SOC in a much smaller scale when using HC, if possible and/ or have several blinded assessors assess the endpoint to estimate the difference of treatment effect between the arms.

**Third**, using simulation, plan for an extensive sensitivity analyses to demonstrate the robustness of the results and to determine the weaknesses or strengths of the analyses by varying assumptions, especially if there is no concurrent control group and the comparison is solely done with HCs [1]. **Fourth**, for any innovative approach, it is highly recommended to include regulatory bodies at the design stage to discuss and explore the potential concerns and issues together. This would increase the quality of the evidence for potential submission in the future, as their familiarity with the approach would help them to coach the scientist properly in the design of the study. **Fifth**, document the nature of both historical and/or concurrent placebo control groups used in your study: Explain why and where these control groups are needed. Explain how the external control groups were chosen and what are the similarities and difference with the current study design and population. Specify how these differences have been addressed in the design and the analyses. Report the results of primary and sensitivity analyses with caution if there is no concurrent control group and the comparison is solely done with HCs.

However, we acknowledge the difficulty of addressing the above points for rare diseases. An alternative option in a rare disease for which none of these resources are available is to prospectively design and implement a well-thought registry or to conduct a natural history study, which may require collaboration with external entities in a larger scale and may delay therapeutic studies. In such cases, it may be preferable to have a randomized therapeutic study than a natural history study in which no one receives therapy. Adaptive designs such as the “informational design” that allow adaptation at the end of the study may be useful for rare diseases where information for designing a therapeutic study may be sparse [19–21].

## Conclusion

FDA is embracing the use of RWD to open up opportunities for more resourceful and innovative approaches, especially in orphan and rare diseases [63]. Consequently, pharmaceutical companies are changing their traditional mindset with development of new hybrid models for integrated decision making. In this evolving era, pharmaceutical and regulatory bodies may be more open to use of HCs as a replacement for or supplement to a concurrent control when a concurrent control is either unethical or impossible, or the condition under study is difficult to enroll. HCs introduce multiple biases compared to a concurrent randomized control. However, these biases may be minimized by careful choice of HCs, design of the study to mimic the HC conditions, and numerous other methods for design, conduct, and analysis of the studies. With careful attention to these approaches, we can apply HCs when they are needed while maximizing scientific validity to the extent feasible.

## Figures and Tables

**Fig. 4:**
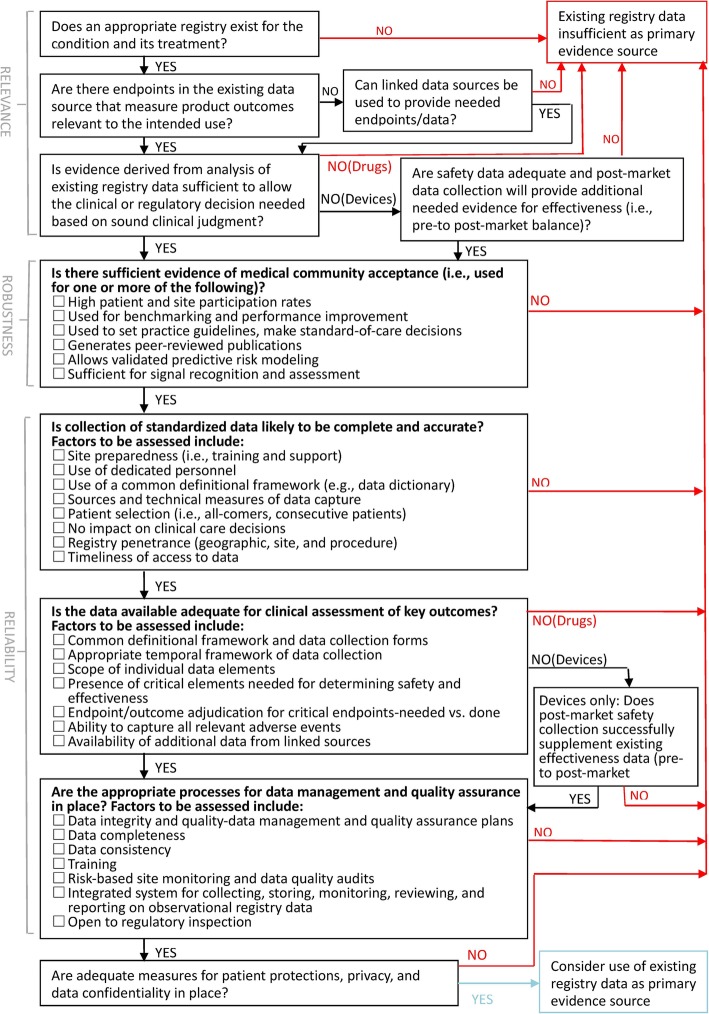
Existing Registry – Historical Assessment

**Fig. 5:**
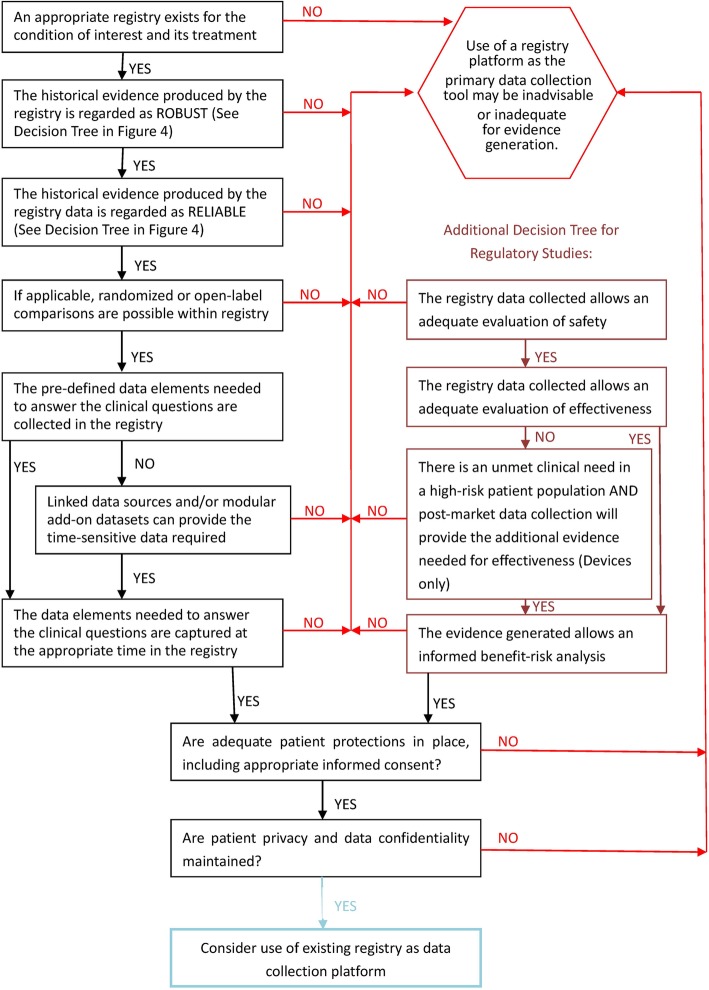
Existing Registry – Suitability Assessment

## Data Availability

Data sharing is not applicable to this article as no datasets were generated or analyzed during the current study.

## Appendix 1

Generally, current RWD data sources have not met our standards below. However, in order to utilize RWD in future clinical designs and in registration trials, we would highly suggest the following as guidance. The relevance of the RWD, RWD sources and resultant analysis is assessed by evaluating several factors as outlined below

- RWD contains sufficient detail to capture the use of the study drug, exposures and the outcome of interest in the appropriate population;
- Data element available for analysis are capable of addressing the specified question when valid and appropriate analytical methods are applied (i.e. the data are amenable to sound clinical and statistical analysis);
- RWD and RWE provided are interpretable using informed clinical / scientific judgement. Important considerations for the assessment of this factor include:
  - Whether the use of the study drug in a real-world population is representative as captured within the data source, and is generalizable to the relevant population being evaluated;
  - Whether the RWD source is used regionally, nationally and/or internationally
  - The overall percentage of patient exposure to the study drug that are captured in the RWD source;
  - The validation protocols and resultant data that are used to evaluate how well the RWD source reflects the patient population’s experience;
  - Whether the RWD study design, study protocol, and/or analysis plan is appropriate to address the regulatory question and capable of being accomplished in a sufficiently timely manner
  - Whether the RWD contains elements to capture specific study drug identification information
  - Whether the RWD adequately captures patient history and preexisting conditions, as well as follow-up information needed evaluate the question being addressed;
  - Whether sufficient data elements are collected to adjust for confounding factors that may impact the exposure or outcome of interest;
  - Whether any linkages performed are sufficiently appropriate and account for differences in coding the reporting across sources
  - The RWD source reporting schedule, including time interval between database close and release, and length of reporting periods;
  - The prior documented use of RWD source for determining outcomes-based quality assessment, validated predictive risk modeling, signal detection, performance improvement benchmarking, and other clinically-meaningful uses;
  - Whether the data elements collected are sufficient for assessing outcomes;
  - Whether supplemental data sources are available and sufficient to provide any missing information or evidence required for any informed decision.

The reliability of RWD, RWD sources, and resultant analyses is assessed by evaluating several factors outlined below. The primary factors include how the data collected (data accrual), whether the people and processes in place during data collection and analysis provide adequate assurance that errors are minimized, and that data quality and integrity are sufficient (data assurance).

- ○ Data Accrual: To ensure the reliability of RWD, the RWD source should have an operational manual or other documentation that pre-specifies the data elements to be collected, data element definitions, methods for data aggregation and documentation, and the relevant time windows for data element collection.
  - ■ The preparedness of individual sites for complete and accurate collection of RWD;
  - ■ Whether a common data capture form was used;
  - ■ Whether a common definitional framework was used;
  - ■ Adherence to a common temporal framework for collection of key data points;
  - ■ The timing of establishing the study plan, protocol, and analysis plan relative to collection or retrieval of RWD;
  - ■ The sources and technical methods used for data element capture;
  - ■ Whether patient selection and enrollment criteria minimize bias and ensure a representative real-world population (e.g. all-come’s design, consecutive patient enrollment);
  - ■ The timeliness of data entry, transmission, and availability
- ○ Data Quality Control: Data quality control is essential for providing confidence in the reliability of RWD and RWE sources. Important factors for considerations include:
  - ■ The quality of data element population;
  - ■ Adherence to source verification procedures and data collection and recording procedures for completeness and consistency;
  - ■ Completeness of data necessary for specified analyses, including adjustment for confounding factors;
  - ■ Data consistency across sites and over time;
  - ■ Evaluation of on-going training programs for data collection and use of data dictionary at participating sites;
  - ■ Evaluation of site and data monitoring practices, if applicable;
  - ■ Use of data quality audit program

## Appendix 2

Clinical Trials Transformation Initiative (CTTI) recommendations to determine if an existing registry is appropriate for embedding clinical trials

- ○ Assess whether the historical evidence generated by an existing registry has demonstrated the reliability, robustness, and relevancy necessary to provide a platform for collecting data in an embedded clinical trial to support regulatory decision-making, with assurance of patient protections (Figs. 4 and 5) [15–17].
  - ■ Data are relevant:
  - ■ Data are adequate in scope and content
  - ■ Data are generalizable: Registry reflects high site and patient participation rates compared with total population
  - ■ Data are robust
  - ■ The registry should be designed to capture reliable data from real-world practice (no protocol-driven treatment)
- ○ Assess if an existing registry contains the elements needed to support a randomized clinical trial. Satisfaction of all the following requirements suggests that the existing registry, together with any appropriate configurable elements, may provide high-quality evidence suitable for regulatory decision-making:
  - ■ Are the data previously generated by the baseline registry historically regarded as robust and reliable (i.e., high-quality data)?
  - ■ Can the baseline registry and its dataset provide the core data needed to answer the question at hand (i.e., relevant or fit for purpose)?
  - ■ Can any processes or data not provided by the baseline registry be added or the registry reconfigured to accommodate these needs (e.g., programming to allow identification of suitable trial participants or documentation of informed consent, modular add-on datasets or linkages to other databases, and appropriate data accessibility with maintenance of patient and data privacy)?

## Appendix 3

Several studies accepted by FDA based on natural history data

- ○ Lysosomal Acid Lipase Deficiency presents a severe to fatal outcome. KANUMA (Sebelipase Alfa) therapy was developed for older children, which is less severe. A large number of very severely affected infants was identified. HC was very useful for approval for infant based on survival analysis and growth failure.
- ○ A disease that causes very brittle bones in infants, children and adults that comes in variety of forms called hypophosphatasia, which would impact how a child would walk. The disease was assessed retrospectively and helped for the approval of a drug called STRENSIQ (Asfotase Alfa).
- ○ Pompe disease is a progressive, multisystemic, debilitating, and often fatal neuromuscular disease characterized by a deficiency of acid alpha-glucosidase (GAA), a lysosomal enzyme. The company subsequently conducted an open-label study of MYOZYME (Alglucosidase Alfa) in patients with Pompe disease because it’s unethical to conduct a double-blind study for patients who were likely to die soon. A total of 62 untreated patients from the natural history study who closely matched the patients in the pivotal trial were identified to serve as a historical control group [64].

A study rejected by FDA based on natural history data:

- ○ A natural history study was undertaken in parallel with a Phase 4 study as a backup for Fabry disease, because a treatment was on the market and the company was concerned that patients randomized to the placebo arm would drop out of the study. The company had suggested to FDA that the patients in the natural history study could serve as a control group. However, FDA did not agree because only a minority of patients in the natural history study qualified for the Phase 4 trial and it was not possible to ensure that patients from the historical database were comparable to patients in the prospective study. FDA was also concerned about the difficulty of adequately determining and adjusting for important baseline characteristics as well as the potential influence of selection bias. In the end, the company was able to conduct its Phase 4 study without using the natural history study patients as a historical control group because patients assigned to the placebo arm stayed in the trial [65].

## Appendix 4

Details on frequentist approaches to analyzing clinical trials with HC

- • Naïve Comparison compares the result of a trial without using historical data with the result of using historical trails side by side without any adjustment for variation among the trials with regards to patient population or the outcome of control arm. It is depreciated as it does not adjust for trial variation; however, it has been used in accepted Health Technology Assessment (HTA) submissions.
- • Benchmarking combines several historical controls, considering within and between study variations, then the distribution of the expected outcomes for a new study for the same treatment could be constructed, and a threshold for the expected outcome can be defined [41].
- • Phase II “pick the winner” different experimental drug is compared with a historical control. No formal statistical comparison between the arms are conducted, and the simple winner of the all arms is the winner of the trial. It is appropriate for comparison of multiple experimental regimens but it is not appropriate for adding an experimental drug to the standard of care [46].
- • Assume drug A has enough evidence that is superior compared to the SOC in a randomized clinical trial. However, you need to show its superiority or non-inferiority to a competing treatment (B) that is already in the market and all that is available is published summary of the treatment B. How can you use this data without conducting another lengthy RCT?
  - ■ Mixed treatment Comparison (MTC) or network analysis is the standard approach. All the data for treatment A and B with a common comparator are collected. The relative effect estimates against the common comparator is obtained, and MTC is performed to derive a summary estimate of A vs. B. However, this method has challenges such as handling heterogeneity between the studies properly.
  - ■ Simulated Treatment Outcome (STC) generates a Prediction model for outcome of treatment A, the simulated outcome for treatment A in trial B is generated using Treatment B summary data, and the result of the treatment A and B are compared. It may introduce biases when the outcome model is not linear such as PFS and OS, binary outcomes, and counts.
  - ■ Matching-adjusted indirect comparison (MAIC) adjusts the population receiving treatment A to match average baseline characteristics of population who received treatment B [41]. Then it compares the outcome across balanced population.
- • Propensity score (PS) is commonly used for adjusting confounding factors, especially in observational studies. PS is the probability of assigning a treatment conditioning on baseline characteristics. It is used to match patients, to stratify patients, to weigh the inverse probability of treatment, or to adjust covariates, so that the treatments groups would be comparable by reducing the selection bias in terms of observed baseline characteristics [42]. Since the matching and stratification approaches are not using PS directly in the analysis, they are less sensitive to misspecification of the PS model. However, King et al. [45], argues that matching based on propensity score often increases imbalance, inefficiency, model dependence, and bias. They suggest that researchers should use other matching methods for example matching based on distance metrics (Malhanobis or Euclidean distance) instead and use PS for many other productive applications such as regression adjustment and inverse weighting.
- • Threshold Crossing is inspired by three concepts of dynamic borrowing using a multi-stage approach, ICH E10 guidelines, and a non-inferiority margin that has to be predetermined before the trial with the help of systematic review of preexisting studies. It introduces a new framework for evidence generation. It defines an estimand by defining the treatment-eligible population, the measurable variable of interest, the measure of intervention effect, and the impact of interfering events such as non-compliance, death, rescue medication, etc. It predefines the agreement on the rules for estimation of the variable of interest in the historical controls. The HCs are selected in an unbiased and the estimation is performed. Using the estimation of efficacy, with the help of expert knowledge and agreement of regulators efficacy and futility thresholds are determined. A single arm trial efficacy is then conducted, blinding the assessors to the endpoints or using several assessors. The impact of unknown and unmeasured potential confounders and assumptions are evaluated by performing sensitivity analysis and a decision for transition to subsequent steps is made [1].
- • Adaptive Design allows adaptations or modifications to different aspects of a trial after its initiation without undermining the validity and integrity of the trial [46].
  - ■ The data from parallel clinical trials could be used to adjust for new findings.
  - ■ Adaptive borrowing based on the evaluation of similarity of historical control and concurrent control based via several interim analysis, and adjusting recruitment for concurrent control
  - ■ Use of an informational cohort within a study to make adaptations to study design in the absence of available natural history data.

Details on Bayesian approaches to analyzing clinical trials with HC:

- • **Meta-analytic** or hierarchical modeling can also be performed using a frequentist approach. However, it is more common in a Bayesian context. It applies dynamic borrowing by placing a distribution across current and historical controls. If the current study is close to the HCs, the estimated variation among the studies will be small. If the current study is far from the HCs, the estimated variation among the studies will be large. Meta-analytic Combined (**MAC**) and Meta-analytic Predictive (**MAP**) are two versions of this approach [34].
- • MAC performs a meta-analysis of historical and current control data, where the estimated parameter is the parameter in the actual trial (no prior is required). MAC is retrospective as the current clinical trial data should be available for the meta-analysis.
- • MAP, on the other hand, performs a meta-analysis of only historical controls and estimates a prior for the parameter of the actual trial (MAP prior), which is combined with the current data (likelihood part) to estimate a posterior to estimate the current clinical trial parameter, using Bayesian analysis. It is prospective as the current clinical trial data is not needed for the meta-analysis and estimation of the prior.
- • In another study using simulations it was shown that methods that estimate parameters for the between-trial heterogeneity generally offer the best trade-off of power, precision and type I error, with the meta-analytic-predictive prior being the most promising method.
- • The results show that it can be feasible to include historical data in the analysis of clinical trials, if an appropriate method is used to estimate the heterogeneity between trials, and the historical data satisfy criteria for comparability [66].
- • When a “MAP prior” is used as the prior in a Bayesian Analysis to estimate the posterior of parameters of interest, then the results of MAP and MAC are the same. This implies that:
  - ○ The result of a Bayesian analysis with prior information give inference on “trial data and HCs data” combined. It is no borrowing of information but a combination of information.
  - ○ The MAC includes always all historical information whereas the MAP and Bayes approach allows controlling the amount of historical information this is included in the inference. Thus, these methods provide a level for reduction of potential bias or detection of bias.
  - ○ Due to vague or non-informative prior for MAC, it will give the same results as a frequentist meta-analysis. This implies that a frequentist MAC incorporates full prior (historical) information as for Bayesian analysis but in a less controlled manner. Accordingly, concerns about introduction of bias by prior information in Bayesian analysis are equivalent for those bias in frequentist MA.
- • Bias-variance model was suggested by Pocock [29]. The difference between past and present was represented by a bias parameter with specified distribution (mean and variance). There are several variations of this model, and it is mostly applied in dose-response and carcinogenicity studies.
- • **Test-then-pool** is a basic form of dynamic borrowing and compares the similarity of historical and concurrent control data with significance level α. If they are similar, it pools the HC and the concurrent control data; otherwise discards HC. In other words, the amount of weight (0 or 1) assigned to the historical data depends on the data in the current trial [37].
- • **Power prior** method estimates an informative prior for the current study. The informative prior is the product of an initial prior (non-informative prior) and the likelihood for the HC data that is raised to a power between 0 and 1 (α_0_). A different power can be specified for each historical control. When alpha is set to 0, HC is 100% discounted, whereas when alpha is set to 1, the HC is pooled without discounting. The resulting ‘posterior’ is used as an informative prior for the current study. The static borrowing version of this method sets α_0_ to a fixed value that may not depend on the similarity of historical and concurrent control data, i.e. set a fixed weight on historical control, i.e. 20%. Alternatively, α_0_ can be set based on the comparison of historical controls and/or the current data. The dynamic borrowing version of this method allows the degree of discounting to be influenced by the difference between the historical and concurrent control data by empirically estimating or placing a ‘vague’ prior distribution on the power parameter [37].
